# Neuronal Localization of SENP Proteins with Super Resolution Microscopy

**DOI:** 10.3390/brainsci10110778

**Published:** 2020-10-25

**Authors:** Luca Colnaghi, Andrea Conz, Luca Russo, Clara A. Musi, Luana Fioriti, Tiziana Borsello, Mario Salmona

**Affiliations:** 1 Department of Molecular Biochemistry and Pharmacology, Istituto di Ricerche Farmacologiche Mario Negri IRCCS, 20156 Milan, Italy; andrea.conz@marionegri.it (A.C.); luca.russo@marionegri.it (L.R.); mario.salmona@marionegri.it (M.S.); 2 Department of Neuroscience, Istituto di Ricerche Farmacologiche Mario Negri IRCCS, 20156 Milan, Italy; claraalice.musi@marionegri.it (C.A.M.); luana.fioriti@marionegri.it (L.F.); tiziana.borsello@unimi.it (T.B.); 3 Department of Pharmacological and Biomolecular Sciences, Milan University, 20133 Milan, Italy

**Keywords:** SUMO, SENPs, neurons, super resolution microscopy

## Abstract

SUMOylation of proteins plays a key role in modulating neuronal function. For this reason, the balance between protein SUMOylation and deSUMOylation requires fine regulation to guarantee the homeostasis of neural tissue. While extensive research has been carried out on the localization and function of small ubiquitin-related modifier (SUMO) variants in neurons, less attention has been paid to the SUMO-specific isopeptidases that constitute the human SUMO-specific isopeptidase (SENP)/Ubiquitin-Specific Protease (ULP) cysteine protease family (SENP1-3 and SENP5-7). Here, for the first time, we studied the localization of SENP1, SENP6, and SENP7 in cultured hippocampal primary neurons at a super resolution detail level, with structured illumination microscopy (SIM). We found that the deSUMOylases partially colocalize with pre- and post-synaptic markers such as synaptophysin and drebrin. Thus, further confirming the presence with synaptic markers of the negative regulators of the SUMOylation machinery.

## 1. Introduction

Synapses are essential structures for the correct function of the nervous tissue [1]. In the vast neuronal proteome, several proteins have the task of modulating synaptic transmission and neuronal excitability [2]. The regulatory activity of these proteins is controlled by post-translational modifications (PTM) [3], which take place both at presynaptic buttons and post-synaptic terminals [4,5,6]. Although SUMOylation has been described as a PTM operating mainly at the nuclear level [7], more recent studies have shown that SUMOylation can also occur outside the nucleus [8], synapses included [9,10]. SUMOylation consists of the covalent addition of members of the small ubiquitin-related modifier (SUMO) family to lysines of target substrates [11]. Mammals express five different variants of SUMO (SUMO 1–5). Although they differ in their aminoacidic sequence, they share an almost identical three-dimensional structure [12]. Similarly to ubiquitin, an enzymatic cascade consisting of E1, E2 and E3 enzymes covalently attaches SUMO1–3 moieties to lysines of target proteins or to other SUMO proteins to form polySUMO chains [7]. SUMOylation of proteins can be reverted by SUMO-specific isopeptidases (SENP1–3 and SENP5–7) [13,14] belonging to the human Ubiquitin-Specific Protease (ULP)/SENP cysteine protease family derived from *Saccharomyces Cerevisiae* Ulp1 and Ulp2 [12,15]. Among them, SENP1–3 and SENP5 are more closely related to Ulp1, while SENP6 and SENP7 are more evolutionary divergent and are more similar to Ulp2 [16]. In cells, SUMO proteases have two main activities: (i) cleavage of immature SUMO precursors to generate mature SUMO and (ii) deconjugation of covalently linked SUMO from target proteins [15]. In vitro studies have shown that SENP1 and SENP2 can target SUMO precursors and have a similar efficiency in the deconjugation of SUMO1 and SUMO2/3 [16]. SENP3 and SENP5 have a preference for SUMO2/3 deconjugation [16,17,18], and SENP6 and SENP7 have a higher affinity for polySUMO2/3 chains [19,20,21,22]. Three additional SUMO-specific isopeptidases, whose role in neurons is still unclear, have been recently identified: DeSumoylating Isopeptidase 1 and 2 (DeSI1 and DeSI2) [23] and Ubiquitin-Specific Protease-Like 1 (USPL1) [24].

Several reports have described the role of SUMOylation machinery in the regulation of some aspects of synaptic function [8,10,25,26]. For instance, SUMO modulates calcium influx and glutamate release [27] and synaptic transmission mediated by kainate receptors [28]. It also targets synaptic proteins such as synapsin 1a [29], the autoreceptor mGluR7 [30], ion channels [31], and several other receptors [26]. However, recently, three reports failed to identify members of the SUMOylation machinery at the synapse [32,33,34,35]. This has led to the need for a better characterization of the neuronal localization of not only SUMO variants [36] but also of other members of the SUMOylation machinery, such as SENPs. Among the SENPs, most of the work on their roles in neurons has been focused on SENP1, SENP3, and SENP6 [25]. SENP1 and SENP6 have been described in dendritic spines, where they colocalize with pre- and post-synaptic markers, such as Bassoon, Homer1, and PSD-95 [37,38]. In primary rat neurons, SENP1 is highly mobile, and the activation of mGluR5 regulates its diffusion within the synapse and the dendrites. The pharmacological stimulation of mGluR5 resulted in a significant reduction in the exit rate of SENP1 from dendritic terminations, translating into a time-dependent accumulation in the post-synaptic sites [39]. In addition, SENPs deregulation can lead to significant morphological and functional changes in the synapse [25]. For example, overexpression of SENP1 induces an increased internalization of the autoreceptor mGluR7, which inhibits the release of glutamate to modulate synaptic plasticity and excitatory neurotransmission [30]. Long-term potentiation (LTP) is also indirectly regulated by SUMO proteases. SENP1 and SENP3, activated by the protein coil-coil and C2 domain-containing 1A (CC2D1A), modulate the SUMOylation and function of Rac1, whose hyperactivity is implicated in synaptic plasticity and cognitive deficits [40]. In light of the recent claims of putative lack of evidence to support the co-localization of the SUMOylation machinery with synaptic markers [32], we analyzed, for the first time with super resolution microscopy, the localization of endogenous SENP1, SENP6 and SENP7 (to represent both Ulp1 and Ulp2 related SUMO isopeptidases) in hippocampal neuronal cultures.

## 2. Materials and Methods

### 2.1. Animals

The procedures requiring the participation of laboratory animals were conducted according to national and international policies and guidelines of the Mario Negri Institute for Pharmacological Research IRCCS. CD1 mice were used to obtain primary neurons.

### 2.2. Primary Cultures

Primary hippocampal neurons were prepared as previously described by Colnaghi et al., 2019 [36]. Briefly, the dissected hippocampi were obtained from two-day-old CD1 mice. The tissues were next incubated at 37 °C for 30 min in solution constituted by 5.8 mM MgCl_2_, 0.5 mM CaCl_2_, 3.2 mM HEPES, 0.2 mM NaOH (pH 7.4, 292 mOsm), and 20 U/mL papain (Sigma). Next, the tissues were mechanically dissociated, and the cells were plated at a concentration of 75,000 cells per well of Ibidi micro-Slide eight well plates. The cultures were maintained in Neurobasal Basal Medium (Gibco) supplemented with B27 (Gibco), penicillin/streptomycin, and 2 mM glutamine.

### 2.3. Immunofluorescence Experiments

Immunofluorescence experiments were performed according to the procedures described in Colnaghi et al. [36]. Primary neurons were fixed and immunolabeled at 12–14 days in vitro (DIV). Fixation was performed in 4% paraformaldehyde (PFA) solution for 15 min, followed by permeabilization with phosphate-buffered saline (PBS), pH 7.4, containing 0.2% Triton X-100 for 1 min. For localization of SENP1, SENP6, and SENP7 proteins, neurons were first blocked for 1 h in PBS containing 1% BSA and next incubated at room temperature for 2 h with primary antibodies in PBS containing 1% BSA and 0.2% Triton X-100. The following antibodies were used: SENP1 (C-12, Santa Cruz, sc-271360), SENP6 (79-M, Santa Cruz, sc-100585), SENP7 (E-8, Santa Cruz, sc-373821), microtubule-associated protein 2 (Map2) (Abcam, AB5392), synaptophsysin (Synaptic System, GmbH101002), and drebrin (Vinci-Biochem, BSR-M05530). Cells were finally incubated with secondary antibodies (DyLight Fluor Antibody, Thermo Fisher Scientific) for 1 h, and Hoechst (Thermo Fisher Scientific, 33342) at the concentration of 2 mg/mL was used to stain nuclei. ProLong Glass Antifade Mountant (Thermo Fisher Scientific) was used as a mounting agent.

### 2.4. Confocal and Structured Illumination Microscopy

Confocal microscopy and structured illumination microscopy (SIM) were performed according to Colnaghi et al., 2019 [36]. In summary: images were obtained using a Nikon N-SIM microscope. Confocal images were acquired with 40× objective. SIM images were collected in 3D-SIM mode. After the acquisition, Fiji (ImageJ) software was used to process the raw images.

### 2.5. Statistical Analysis

JACoP [41], a toolbox for subcellular co-localization analysis under ImageJ, was used to conduct co-localization analysis to evaluate overlapping fluorescence signals. We analyzed 45 SIM images—taken from three different experiments—for each condition. The overlap of signals was calculated with Pearson’s correlation coefficients. To evaluate the reciprocal co-localization between SENP1, SENP6, and SENP7 and synaptic markers synaptophysin or drebrin, we calculated the parameters Mander’s M1 and M2 [42], setting a manual threshold to avoid background noise. Finally, the graphs were obtained using GraphPad Prism 7.

## 3. Results

### 3.1. SENP1 Partially Colocalizes with the Presynaptic Marker Synaptophysin and with the Postsynaptic Marker Drebrin

**Figure 1:**
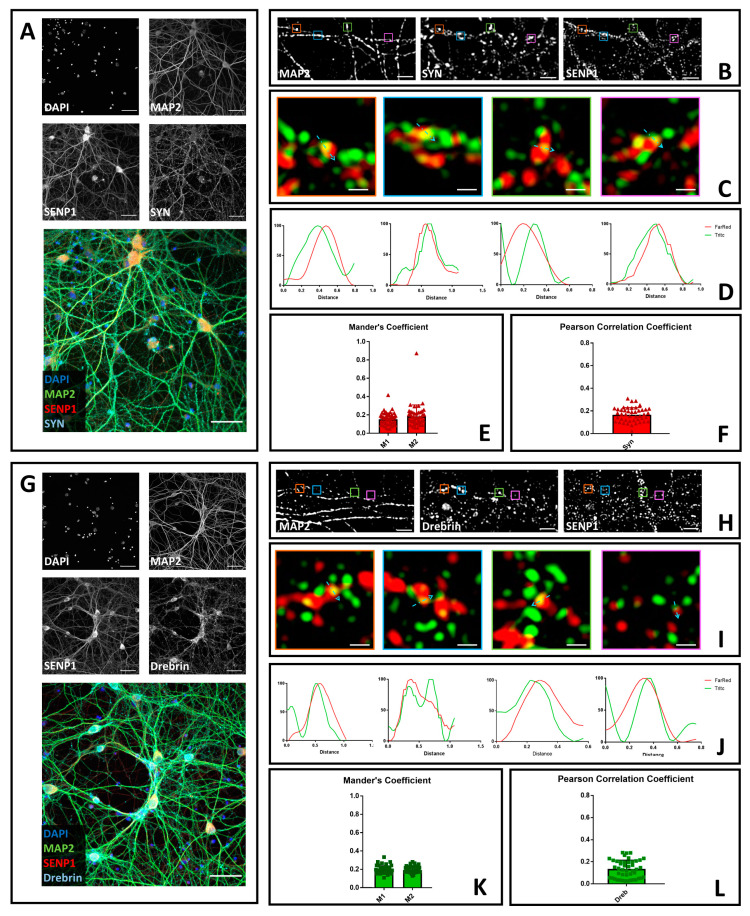
Confocal and structured illumination microscopy (SIM) microscopy to determine the neuronal localization of SENP1. (**A**) Neurons were fixed and stained with anti-SENP1 (red), anti-synaptophysin (light blue) and anti-microtubule-associated protein 2 (Map2) (green). Images were obtained using a Nikon N-SIM confocal microscope and overlaid to assess protein localization. Nuclei were stained with Hoechst. Scale bar of 50 μm. (**B**) 3D-SIM images acquired with a 100× objective. Scale bar of 2 μm. Squares represent the location of the inset present in panel C. (**C**) Merge images of reconstructed 3D-SIM images of SENP1 (green) and synaptophysin (red). Scale bar of 0.5 μm. (**D**) Intensity profile (green for SENP1 and red for synaptophysin) representing the values indicated by the light blue arrow in panel C. The values were normalized to 100 (arbitrary unit). (**E**) Mander’s coefficients of SENP1 co-localization with synaptophysin (M1) and synaptophysin co-localization with SENP1 (M2). (**F**) Pearson’s correlation coefficient of SENP1 and synaptophysin (SYN). Data are the mean ± SD of 45 fields from three independent experiments. (**G**) Neurons were fixed and stained with anti-SENP1 (red), anti-drebrin (light blue), and anti-Map2 (green). Images were obtained using a Nikon N-SIM confocal microscope and overlaid to assess protein localization. Nuclei were stained with Hoechst. Scale bar of 50 μm. (**H**) 3D-SIM images acquired with a 100× objective. Scale bar of 2 μm. Squares represent the location of the inset present in panel C. (**I**) Merge images of reconstructed 3D-SIM images of SENP1 (green) and drebrin (red). Scale bar of 0.5 μm. (**J**) Intensity profile (green for SENP1 and red for drebrin) representing the values indicated by the light blue arrow in panel C. The values were normalized to 100 (arbitrary unit). (**K**) Mander’s coefficients of SENP1 co-localization with drebrin (M1) and drebrin co-localization with SENP1 (M2). (**L**) Pearson’s correlation coefficient of SENP1 and drebrin (Dreb). Data are the mean ± SD of 45 fields from three independent experiments.

To determine whether SENP1 colocalizes with synaptic markers, we cultured primary hippocampal neurons. Upon maturation, we fixed the cells and stained them with antibodies against SENP1, MAP2, a neuronal marker to identify neuronal processes, and synaptophysin, a presynaptic marker [43]. We next acquired images, using confocal microscopy and a 40× objective, to visualize the morphology and the staining of the neurons, confirming a nuclear and cytoplasmic localization for SENP1 [44] (Figure 1A). Next, we identified an area of neuronal processes and performed 3D-SIM (Figure 1B) [45,46]. This technique bypasses the diffraction limits of optical microscopes to reach a resolution of about 100 nm laterally to match the size of synapses [47,48,49,50]. Profile analysis of single loci suggests that SENP1 and synaptophysin colocalize in neuronal processes (Figure 1C,D). To confirm the co-localization results, we decided to perform a similar experiment using a second SENP1 primary antibody. Similarly to our first analysis, we found that SENP1 can localize with synaptophysin (Supplemental Figure S1A). To quantify the co-localization between SENP1 and synaptophysin, we processed the images to obtain the fractions of SENP1 that overlap with synaptophysin signal (Mander’s M1) and vice versa (Mander’s M2) [51]. We obtained values between 0 and 0.5 which suggest a low-to-medium co-localization between the two proteins (Figure 1E). Next, we calculated Pearson’s correlation coefficient, which, with values above 0 and below 0.4, confirmed the partial presynaptic co-localization of SENP1 (Figure 1F). Overall, the data confirm the co-localization of SENP1 and presynaptic markers [39].

To determine whether SENP1 also localizes with post-synaptic markers, we co-stained neurons with antibodies against SENP1, MAP2, and the post-synaptic marker drebrin [52] (Figure 1G–I). Intensity profiles of 3D-SIM images suggest that, in neuronal processes, SENP1 can also localize with drebrin (Figure 1J). We confirmed the results using a second antibody against SENP1 (Supplemental Figure S1B). Finally, to generalize the findings, we quantified the co-localization by analyzing the Mander’s coefficients and Pearson’s correlation coefficients, the values of which also confirmed a low-to-medium co-localization between SENP1 and drebrin (Figure 1K,L).

### 3.2. SENP6 Partially Colocalizes with the Presynaptic Marker Synaptophysin and with the Postsynaptic Marker Drebrin

**Figure 2:**
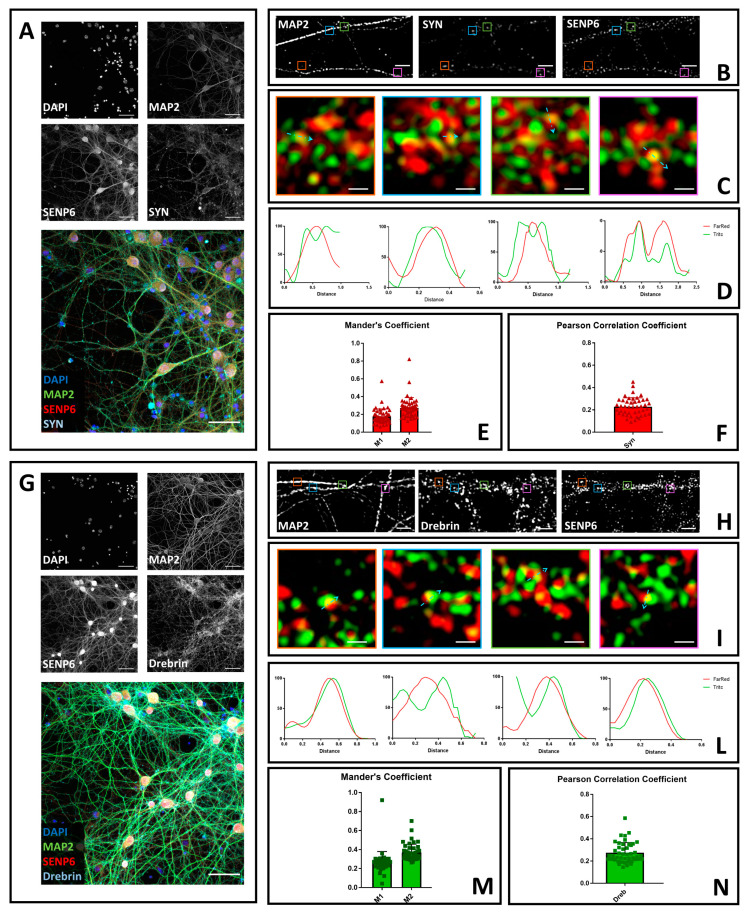
Confocal and SIM microscopy to determine the neuronal localization of SENP1. (**A**) Neurons were fixed and stained with anti-SENP6 (red), anti-synaptophysin (light blue), and anti-Map2 (green). Images were obtained using a Nikon N-SIM confocal microscope and overlaid to assess protein localization. Nuclei were stained with Hoechst. Scale bar of 50 μm. (**B**) 3D-SIM images acquired with a 100× objective. Scale bar of 2 μm. Squares represent the location of the inset present in panel C. (**C**) Merge images of reconstructed 3D-SIM images of SENP6 (green) and synaptophysin (red). Scale bar of 0.5 μm. (**D**) Intensity profile (green for SENP6 and red for synaptophysin) representing the values indicated by the light blue arrow in panel C. The values were normalized to 100 (arbitrary unit). (**E**) Mander’s coefficients of SENP6 co-localization with synaptophysin (M1) and synaptophysin co-localization with SENP6 (M2). (**F**) Pearson’s Correlation Coefficient of SENP1 and synaptophysin (SYN). Data are the mean ± SD of 45 fields from three independent experiments. (**G**) Neurons were fixed and stained with anti-SENP6 (red), anti-drebrin (light blue), and anti-Map2 (green). Images were obtained using a Nikon N-SIM confocal microscope and overlaid to assess protein localization. Nuclei were stained with Hoechst. Scale bar of 50 μm. (**H**) 3D-SIM images acquired with a 100× objective. Scale bar of 2 μm. Squares represent the location of the inset present in panel C. (**I**) Merge images of reconstructed 3D-SIM images of SENP6 (green) and drebrin (red). Scale bar of 0.5 μm. (**J**) Intensity profile (green for SENP6 and red for drebrin) representing the values indicated by the light blue arrow in panel C. The values were normalized to 100 (arbitrary unit). (**K**) Mander’s coefficients of SENP6 co-localization with drebrin (M1) and drebrin co-localization with SENP6 (M2). (**L**) Pearson’s Correlation Coefficient of SENP6 and drebrin (Dreb). Data are the mean ± SD of 45 fields from three independent experiments.

Next, we performed a similar analysis for SENP6. For its localization, we co-stained cultured hippocampal neurons with antibodies against SENP6, MAP2, and synaptophysin. We first acquired images with confocal microscopy and a 40× objective (Figure 2A) and subsequently switched to 3D-SIM (Figure 2B,C). With this first analysis, we confirmed that SENP6 is mainly a nuclear protein that was also localized to the cytoplasm. We next analyzed the co-localization between SENP6 and synaptophysin with profile analysis using two different antibodies (Figure 2D and Supplemental Figure S2A). We quantified the co-localization by Mander’s coefficients and Pearson’s correlation coefficients, and we obtained, for both, values between 0 and 0.6, to suggest a partial overlap of signals (Figure 2E,F). We next performed a similar analysis to determine the co-localization of SENP6 with drebrin. We started with confocal microscopy (Figure 2G); next, we performed 3D-SIM analysis (Figure 2H,I and Supplemental Figure S2B) to determine the profile analysis of co-localization (Figure 2J) and Mandel’s and Pearson’s coefficients (Figure 2K,L). We obtained values similar to SENP6 and synaptophysin co-localization. Overall, these data indicate the co-localization of SENP6 with both presynaptic and post-synaptic markers.

### 3.3. SENP7 Partially Colocalizes with the Presynaptic Marker Synaptophysin and with the Postsynaptic Marker Drebrin

**Figure 3:**
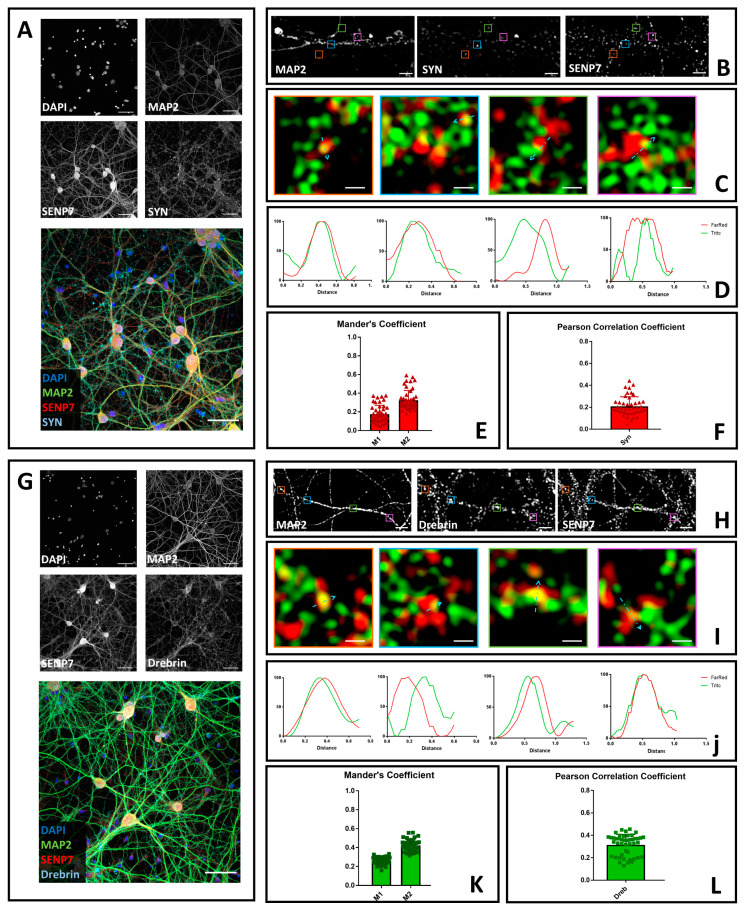
Confocal and SIM microscopy to determine the neuronal localization of SENP7. (**A**) Neurons were fixed and stained with anti-SENP7 (red), anti-synaptophysin (light blue) and anti-Map2 (green). Images were obtained using a Nikon N-SIM confocal microscope and overlaid to assess protein localization. Nuclei were stained with Hoechst. Scale bar of 50 μm. (**B**) 3D-SIM images acquired with a 100× objective. Scale bar of 2 μm. Squares represent the location of the inset present in panel C. (**C**) Merge images of reconstructed 3D-SIM images of SENP7 (green) and synaptophysin (red). Scale bar of 0.5 μm. (**D**) Intensity profile (green for SENP7 and red for synaptophysin) representing the values indicated by the light blue arrow in panel C. The values were normalized to 100 (arbitrary unit). (**E**) Mander’s coefficients of SENP7 co-localization with synaptophysin (M1) and synaptophysin co-localization with SENP7 (M2). (**F**) Pearson’s Correlation Coefficient of SENP7 and synaptophysin (SYN). Data are the mean ± SD of 45 fields from three independent experiments. (**G**) Neurons were fixed and stained with anti-SENP7 (red), anti-drebrin (light blue), and anti-Map2 (green). Images were obtained using a Nikon N-SIM confocal microscope and overlaid to assess protein localization. Nuclei were stained with Hoechst. Scale bar of 50 μm. (**H**) 3D-SIM images acquired with a 100× objective. Scale bar of 2 μm. Squares represent the location of the inset present in panel C. (**I**) Merge images of reconstructed 3D-SIM images of SENP7 (green) and drebrin (red). Scale bar of 0.5 μm. (**J**) Intensity profile (green for SENP7 and red for drebrin) representing the values indicated by the light blue arrow in panel C. The values were normalized to 100 (arbitrary unit). (**K**) Mander’s coefficients of SENP7 co-localization with drebrin (M1) and drebrin co-localization with SENP7 (M2). (**L**) Pearson’s Correlation Coefficient of SENP7 and drebrin (Dreb). Data are the mean ± SD of 45 fields from three independent experiments.

Lastly, we decided to determine the neuronal localization of SENP7. Differently from SENP1 and SENP6, SENP7 has only been marginally studied in neurons. Therefore, our analysis has the potential to improve our understanding of the SUMOylation machinery in this context. To this end, we stained cultured hippocampal neurons with antibodies directed against SENP7, MAP2, and the synaptic markers drebrin and synaptophysin. We acquired images using both confocal microscopy with a 40× objective (Figure 3A,G) and 3D-SIM microscopy (Figure 3B,C,H,I and Supplemental Figure S3A,B). Similarly to SENP1 and SENP6, we found that SENP7 is both a nuclear and cytoplasmic protein. Outside the nucleus, it partially colocalizes with synaptic markers, as indicated by Mander’s and Pearson’s correlation coefficients (Figure 3D–F,J–L).

Together, these results reinforce the hypothesis of the co-localization of several components of the SUMOylation machinery with synaptic markers.

## 4. Discussion

In the past decade, several reports have described how SUMOylation machinery plays key roles in the function and homeostasis of synapses [9,25,26]. A recent set of papers, however, failed to detect members of the SUMOylation machinery with synaptic markers [21,22,23], urging a more in-depth analysis of the neuronal localization of SUMO related proteins. In our previous study, we confirmed using SIM the co-localization of SUMO1, SUMO2/3, and the E2 SUMOylation enzyme Ubc9 with pre- and post-synaptic markers [36]. In this study, we focused on the ULP/SENP family of proteases, a class of enzymes that regulate the dynamic balance between conjugated and de-conjugated SUMO. Among the members of the enzymatic family, we chose to study SENP1, SENP6, and SENP7, and we analyzed their neuronal localization with SIM. As expected, we found that the three enzymes are both nuclear and cytosolic proteins.

Quantitative analysis indicates that SENP1 colocalizes with synaptophysin and drebrin with a partial overlap between the proteins. These data are in agreement with past observations on the regulation of SENP1 by mGluR5, whose activation induces accumulation of the protease in dendritic spines [39]. There, SENP1 acts synergistically with Ubc9 to regulate synaptic homeostasis [39]. Moreover, in primary neurons, SENP1 also regulates the degree of SUMOylated mGluR7 to modulate its internalization [30]. Finally, in the rat brain, SENP1, together with SENP6, is highly expressed in the initial stages of development and localizes to both the nucleus and cytoplasm. Its expression decreases in adulthood [38]. Differently from SENP1, the neuronal localization and function of SENP6 and SENP7 have been less explored. However, similarly to SENP1, we found that SENP6 and SENP7 partially colocalize with synaptophysin and drebrin, thus suggesting the existence in neuronal processes of polySUMOchains, which are their preferred SUMO substrates. SUMO2/3 chains have yet to be characterized in neurons. Still, they could regulate several aspects of synaptic function, such as degradation of target proteins [53], an essential process in memory formation and recall [54].

## 5. Conclusions

In conclusion, using super resolution microscopy, we examined the cellular localization of SENP proteins in primary cultures of murine hippocampal neurons. We found that SENP1, SENP6 and SENP7 are both nuclear and cytoplasmic proteins that can colocalize with synaptic markers such as drebrin and synaptophysin. We therefore confirmed previous evidence that suggest a major role of the SUMOylation machinery in the physiology of neurons and synapses.

## Supplementary Materials

The following are available online at https://www.mdpi.com/2076-3425/10/11/778/s1, Figure S1: SIM microscopy to determine the neuronal localization of SENP1 using a second antibody (OriGene, TA800001). Figure S2: SIM microscopy to determine the neuronal localization of SENP6 using a second antibody (Novus Biologicals, H00026054-M01). Figure S3: SIM microscopy to determine the neuronal localization of SENP7 using a second antibody (Novus Biologicals, H00057337-M01).
