# Supplementary material for: Neuronal Localization of SENP Proteins with Super Resolution Microscopy
Source: Brain Sci. 2020 Oct 25;10(11):778. doi: 10.3390/brainsci10110778 (PMC7693135; doi:10.3390/brainsci10110778)
Supplement: Supplementary file 1 [file brainsci-10-00778-s001.pdf]

brain sciences

MDPI

SUPPLEMENTAL FIGURE 1

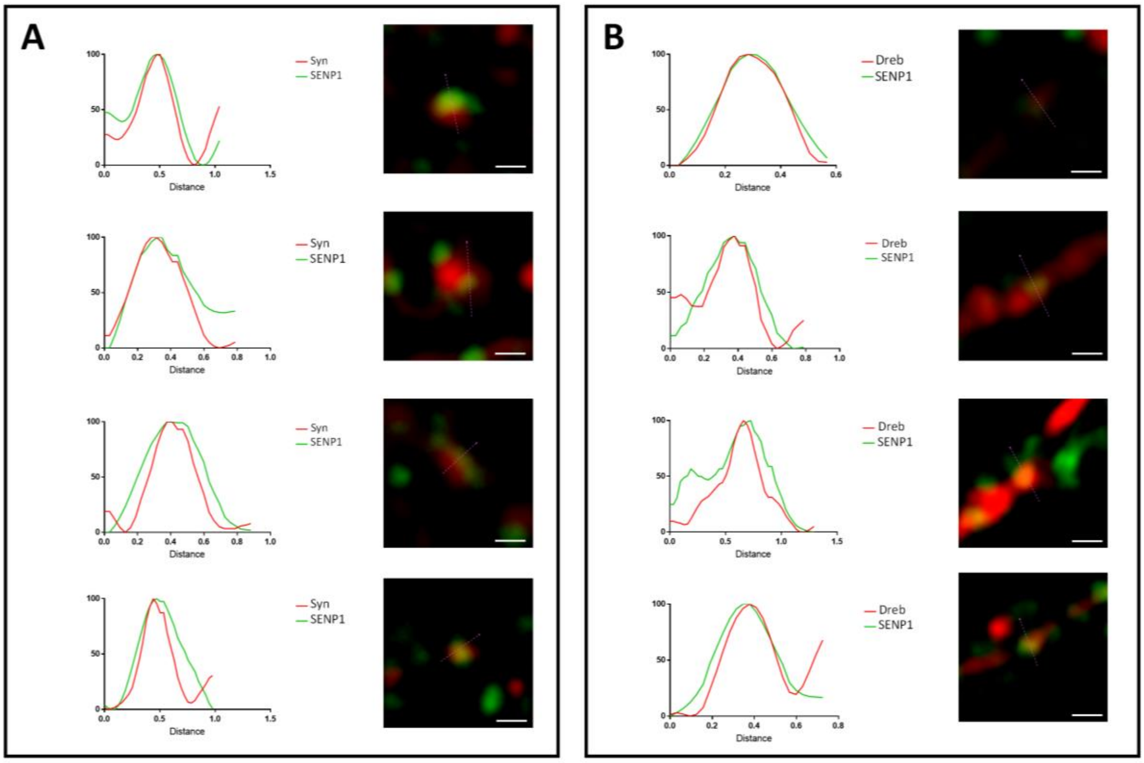

Figure S1: SIM microscopy to determine the neuronal localization of SENP1 using a second antibody (OriGene, TA800001). (A) Neurons were fixed and stained with anti-SENP1, anti-synaptophysin and anti-Map2 to identify neuronal processes. 3D-SIM images were obtained using a Nikon N-SIM confocal microscope 100× objective and overlaid to assess protein localization. Merge images of reconstructed 3D-SIM images of SENP1 (green) and synaptophysin (red). Scale bar of 0.5 μm. Intensity profile (green for SENP1 and red for synaptophysin) representing the values indicated by the purple arrow in the squares. The values were normalized to 100 (arbitrary unit). (B) Neurons were fixed and stained with anti-SENP1, anti-drebrin and anti-Map2 to identify neuronal processes. 3D-SIM images were obtained using a Nikon N-SIM confocal microscope 100× objective and overlaid to assess protein localization. Merge images of reconstructed 3D-SIM images of SENP1 (green) and synaptic drebrin (red). Scale bar of 0.5 μm. Intensity profile (green for SENP1 and red for drebrin) representing the values indicated by the purple arrow in the squares. The values were normalized to 100 (arbitrary unit).

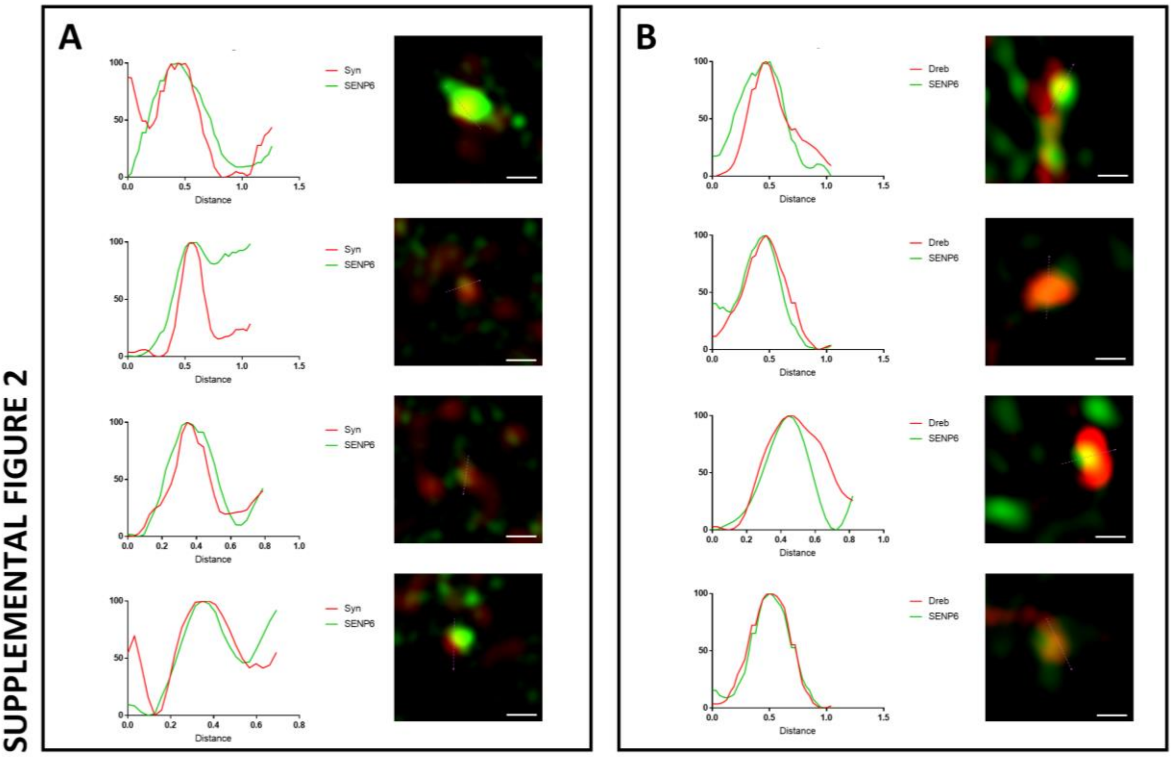

Figure S2: SIM microscopy to determine the neuronal localization of SENP6 using a second antibody (Novus Biologicals, H00026054-M01). (A) Neurons were fixed and stained with anti-SENP6, anti-synaptophysin and anti-Map2 to identify neuronal processes. 3D-SIM images were obtained using a Nikon N-SIM confocal microscope 100× objective and overlaid to assess protein localization. Merge images of reconstructed 3D-SIM images of SENP6 (green) and synaptophysin (red). Scale bar of 0.5 µm. Intensity profile (green for SENP6 and red for synaptophysin) representing the values indicated by the purple arrow in the squares. The values were normalized to 100 (arbitrary unit). (B) Neurons were fixed and stained with anti-SENP6, anti-drebrin and anti-Map2 to identify neuronal processes. 3D-SIM images were obtained using a Nikon N-SIM confocal microscope 100× objective and overlaid to assess protein localization. Merge images of reconstructed 3D-SIM images of SENP6 (green) and synaptic drebrin (red). Scale bar of 0.5 µm. Intensity profile (green for SENP6 and red for drebrin) representing the values indicated by the purple arrow in the squares. The values were normalized to 100 (arbitrary unit).

SUPPLEMENTAL FIGURE 3

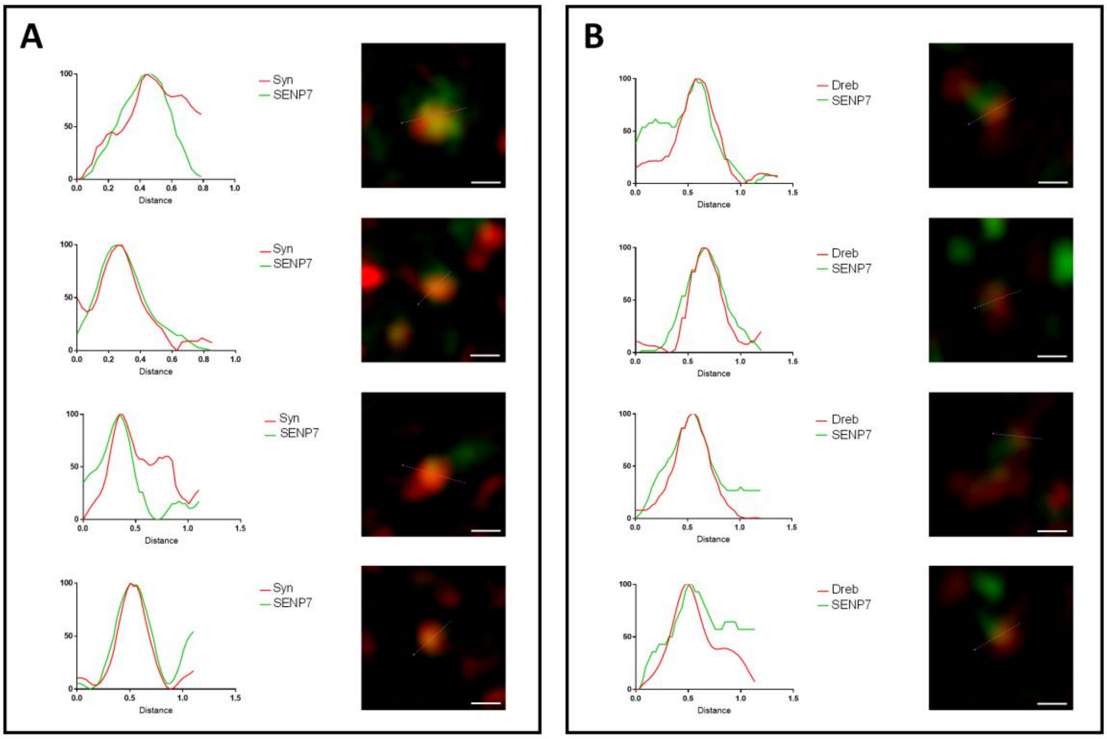

Figure S3: SIM microscopy to determine the neuronal localization of SENP7 using a second antibody (Novus Biologicals, H00057337-M01). (A) Neurons were fixed and stained with anti-SENP7, anti-synaptophysin and anti-Map2 to identify neuronal processes. 3D-SIM images were obtained using a Nikon N-SIM confocal microscope 100× objective and overlaid to assess protein localization. Merge images of reconstructed 3D-SIM images of SENP7 (green) and synaptophysin (red). Scale bar of 0.5 µm. Intensity profile (green for SENP7 and red for synaptophysin) representing the values indicated by the purple arrow in the squares. The values were normalized to 100 (arbitrary unit). (B) Neurons were fixed and stained with anti-SENP7, anti-drebrin and anti-Map2 to identify neuronal processes. 3D-SIM images were obtained using a Nikon N-SIM confocal microscope 100× objective and overlaid to assess protein localization. Merge images of reconstructed 3D-SIM images of SENP7 (green) and synaptic drebrin (red). Scale bar of 0.5 µm. Intensity profile (green for SENP7 and red for drebrin) representing the values indicated by the purple arrow in the squares. The values were normalized to 100 (arbitrary unit).
